# The Gram-negative phytopathogen *Xanthomonas campestris* pv. *campestris* employs a 5'UTR as a feedback controller to regulate methionine biosynthesis

**DOI:** 10.1099/mic.0.000690

**Published:** 2018-07-19

**Authors:** Jian-Ling Zhang, Dan Wang, Yu-Wei Liang, Wan-Ying Zhong, Zhen-Hua Ming, Dong-Jie Tang, Ji-Liang Tang

**Affiliations:** State Key Laboratory for Conservation and Utilization of Subtropical Agro-bioresources, College of Life Science and Technology, Guangxi University, 100 Daxue Road, Nanning, Guangxi 530004, PR China

**Keywords:** *Xanthomonas*, methionine biosynthesis, 5’UTR, post-transcriptional regulation, virulence

## Abstract

The synthesis of methionine is critical for most bacteria. It is known that cellular methionine has a feedback effect on the expression of *met* genes involved in *de novo* methionine biosynthesis. Previous studies revealed that Gram-negative bacteria control *met* gene expression at the transcriptional level by regulator proteins, while most Gram-positive bacteria regulate *met* genes at post-transcriptional level by RNA regulators (riboregulators) located in the 5′UTR of *met* genes. However, despite its importance, the methionine biosynthesis pathway in the Gram-negative *Xanthomonas* genus that includes many important plant pathogens is completely uncharacterized. Here, we address this issue using the crucifer black rot pathogen *Xanthomonas campestris* pv. *campestris* (*Xcc*), a model bacterium in microbe–plant interaction studies. The work identified an operon (*met*) involved in *de novo* methionine biosynthesis in *Xcc*. Disruption of the operon resulted in defective growth in methionine-limited media and *in planta*. Western blot analysis revealed that the expression of the operon is dependent on methionine levels. Further molecular analyses demonstrated that the 5′UTR, but not the promoter of the operon, is involved in feedback regulation on operon expression in response to methionine availability, providing an example of a Gram-negative bacterium utilizing a 5′UTR region to control the expression of the genes involved in methionine biosynthesis.

## Introduction

To maintain regular protein synthesis, bacterial cells require a constant supply of amino acids. Most achieve this by taking up amino acids from the surrounding environment and/or synthesizing amino acids from simpler compounds (i.e. *de novo* synthesis). The synthesis of amino acids is considered a complicated and biologically expensive process and is therefore strictly regulated. It is known that among the efficient mechanisms of regulation is feedback control at the transcriptional or post-transcriptional level [1]. Of the 20 standard amino acids which are needed for bacterial cell viability, the sulfur-containing methionine [(formyl-)methionine] is somewhat unique. This is because it is the first amino acid at the N-terminus of most proteins and plays an irreplaceable role in the initiation of protein biosynthesis. In addition, the methionine derivative S-adenosylmethionine (SAM) serves as a universal methyl group donor in a variety of methyltransferase reactions [2].

**Fig. 1.**
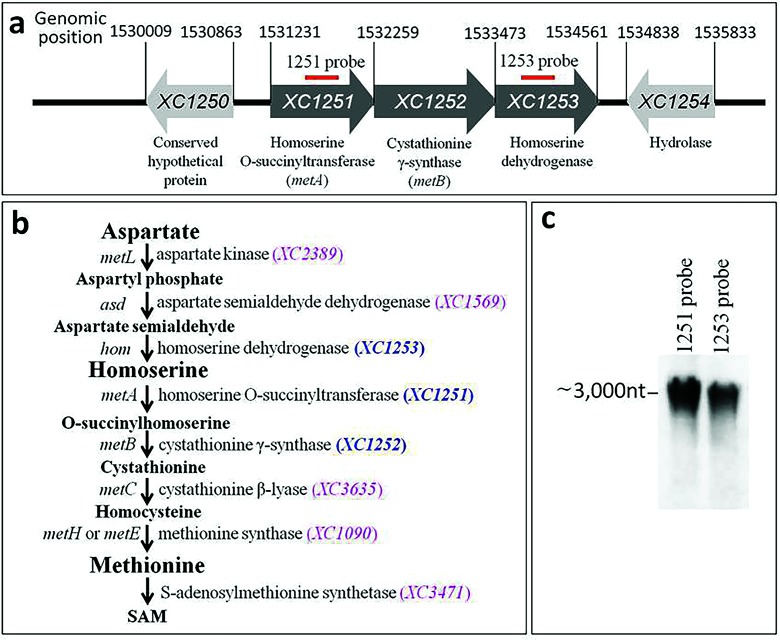
Genetic organization and identification of *Xcc met* operon. (a) A schematic diagram showing the genetic organization of the *met* operon (*XC1251-XC1252-XC1253*). The sequences matched to the probes used in the Northern blotting analysis are indicated by thick, red lines above the genes. (b) Predicted methionine biosynthesis pathway in *Xcc*. (c) Detection of transcripts of the *met* operon by Northern blotting.

Most bacterial species, with the exception of some endosymbionts, are able to synthesize methionine using the trans-sulfuration pathway and/or the direct sulfhydrylation pathway from homoserine [3]. The trans-sulfuration pathway in *Escherichia coli* has been extensively characterized as having four steps: homoserine → O-succinyltransferase → cystathionine → homocysterine → methionine, which are catalysed by homoserine O-succinyltransferase (encoded by *metA*), cystathionine γ-synthase (encoded by *metB*), cystathionine β-lyase (encoded by *metC*) and methionine synthase (encoded by *metH* or *metE*), respectively [4] (Fig. 1). Furthermore, it is defined that the homoserine is derived from aspartate in three steps: aspartate → aspartyl phosphate → aspartate semialdehyde → homoserine, which are catalysed by bi-functional aspartate kinase/homoserine dehydrogenase (encoded by *metL*), aspartate-β-semialdehyde dehydrogenase (encoded by *asd*) and homoserine dehydrogenase (encoded by *hom*), respectively [5] (Fig. 1).

The way in which methionine biosynthesis is regulated has also been characterized in certain Gram-negative and -positive bacteria. The general principle for this is similar to the biosynthesis of most other amino acids, where methionine biosynthesis is inactive when methionine is available in the surroundings and active when methionine is limited. The availability of cellular methionine has an effect on the expression of the genes involved in methionine biosynthesis (most of these were called *met* genes). However, the detailed molecular basis of *met* gene regulation differs among bacteria, most likely due to the environment in whichthey are found. In general, Gram-positive bacteria utilize RNA regulators (riboregulators) that function at the post-transcriptional level, for example the S-box and T-box riboswitches. Conversely, Gram-negative bacteria employ protein regulators that function at the transcriptional level [6–9].

Although extensive studies have been carried out on *E. coli* and some other Gram-negative bacteria [4, 10], little is known about methionine biosynthesis and its regulation in Gram-negative phytopathogenic bacteria [11–13]. *Xantomonas campestris* pv. *campestris* (*Xcc*) is a member of the large genus *Xanthomonas*, which comprises 27 species of Gram-negative bacteria, most of which are plant pathogens. As well as being an important plant pathogen, *Xcc* is considered a model pathogen for studying the molecular basis in microbe–plant interactions [14]. In the current study, we reconstructed the methionine biosynthesis pathway in an *Xcc* strain by integrating the genome information from *Xcc* and what is known regarding the *E. coli* methionine biosynthesis route. Using this information, we demonstrated that the synthesis of methionine is critical for *Xcc* to attain regular growth and full virulence in host plant tissues. Additionally, we identified the *met* operon consisting of three open reading frames (ORFs) that encode enzymes potentially involved in catalysing methionine biosynthesis. More importantly, we obtained direct evidence showing that the 5′UTR, but not the promoter of the *met* operon, is involved in feedback regulation of its expression in response to methionine availability. To the best of our knowledge, this provides the first example of a Gram-negative bacterium using 5′UTR to regulate methionine biosynthesis.

## Methods

### Bacterial strains, plasmids and growth conditions

The bacterial strains and plasmids used in this work are listed in Table S1 (available in the online version of this article). *Xcc* and *E. coli* strains were grown under conditions described previously [15].

### Construction of the mutant strain 1201PK2

The mutant 1201PK2 was constructed by the homologous suicide plasmid integration method as described by Windgassen and associates [16]. A 392 bp internal fragment of *XC1251* was amplified using the DNA of the *Xcc* wild-type strain 8004 as template and the primer pair 1251 M-F/1251 M-R (Table S2). The DNA fragment was cloned into the suicide plasmid pK18mob, creating pK1251, which was introduced into strain 8004 by conjugation. The mutant transconjugants obtained with an insertion in *XC1251* were confirmed by PCR using the primer pair P18con-F/1251con-R (Table S2). One of the confirmed mutants, named 1201PK2, was used for further study.

### Determination of the transcription start site (TSS) of the *met* operon (*XC1251-XC1253*) by 5′-RACE

Total RNA was extracted from the wild-type strain 8004 and treated with DNase I. Five micrograms of the DNA-free RNA and 15 pmol of the gene-specific primer 1251GSP1 (CAACCAGCGTGTGCAGAC) were incubated at 70 °C for 5 min and then 42 °C for 1 h in the presence of 1×M-MuLV-RT buffer, 1 mM dNTPs, 20 U RNase inhibitor and 200 U M-MuLV Reverse Transcriptase. After treatment with 2 U µl^−1^ RNase H for 30 min (to remove the remaining RNAs), the reaction product (cDNA) was purified using S.N.A.P.Column and finally resolved in 50 µl sterilized dH_2_O. Then, 10 µl purified cDNA was incubated at 37 °C for 10 min in the presence of 0.2 mM dCTP and 20 U of TDT (terminal deoxynucleotidyl transferase) to add a poly(C) tail to the 3′-end of the cDNA. Finally, 5 µl poly(C) tailed cDNA was used as template for PCR. To improve sensitivity, two rounds of semi-nested PCR were performed. The first of these was performed using 5 µl poly(C) tailed cDNA as template, and AAP (GGCCACGCGTCGACTAGTACGGGIIGGGIIGGGIIG) and an internal oligonucleotide, 1251GSP2 (CGCCGCATGTCGGCTGGCGAATTGC) as primers; the second was performed using the product from the first PCR as template, and AUAP (GGCCACGCGTCGACTAGTAC) and another internal oligonucleotide, 1251GSP3 (ATGCCGCCCGCCACGAACACCAC) as primers. After gel purification, the final PCR products were cloned into the pMD18-T vector and sequenced.

### Construction of strain XC1251-3F expressing the recombinant protein XC1251-3FLAG

The strain XC1251-3F (Table S1) expressing a recombinant XC1251 protein (XC1251-3FLAG) with a 3×Flag tag at its C-terminus was constructed by in-frame insertion of the 3×Flag-coding sequence into the 3′-end (just before the stop codon TGA) of the XC1251 coding sequence in strain 8004, using the suicide vector-mediated unmarked allelic replacement method described by Patey *et al*. [17]. Briefly, a 781 bp DNA upstream flanking sequence of *XC1251* and a 607 bp DNA fragment containing the 3×Flag-coding sequence, the stop codon TGA and downstream flanking sequence of XC1251 were generated by PCR amplification using the DNA of strain 8004 as template and the primer pairs 1251-3F-U-F/1251-3F-U-R and 1251–3F-D-F/1251–3F-D-R (Table S2), respectively. The DNA fragments thus obtained were linked by fusion PCR and cloned into the suicide plasmid pK18mobsacB, generating the recombinant plasmid pK1251-3F, which was then transferred into strain 8004 by conjugation. Single-crossover integration transconjugants were selected and confirmed by sucrose-sensitive phenotype. A confirmed single-crossover transconjugant was checked for the in-frame insertion of 3×Flag-coding sequence before the stop codon of *XC1251* by sequencing analysis of the PCR products generated by amplification of its genomic DNA with the primer pair 1251-3F-U-F/1251-3F-D-R (Table S2). The expression of XC1251-3FLAG protein was further confirmed by Western blotting.

### Construction of promoter-*gusA* and 5′UTR-*gusA* fusion reporters

Two promoter-*gusA* transcriptional fusion reporter plasmids, named pP*1251*L and pP*1251*S, were constructed by fusing 109 and 52 bp DNA sequences upstream of the TSS with the 1826 bp DNA segment of the promoterless but SD-containing *gusA* gene of *E. coli*, respectively, and cloning the 1935 and 1878 bp fused DNA fragments thus obtained into the promoterless cloning sites of the vector pLAFR6, respectively. The 1935 and 1878 bp fused DNA fragments were obtained by PCR amplification using *E. coli* K12 genomic DNA as template and the primer pairs P1251L-SDgusA-F/gusA-R and P1251S-SDgusA-F/gusA-R (Table S2), respectively.

The 5′UTR of *XC1251-XC1253* operon-*gusA* transcriptional fusion reporter p5UTR-SD^+^ and translational fusion reporter p5UTR-SD^-^ were constructed by cloning the Plac-5′UTR-SD^+^*gusA* and Plac-5′UTR-SD^-^*gusA* fragment into the vector pLAFR6, respectively. Plac-5′UTR-SD^+^*gusA* and Plac-5′UTR-SD^-^*gusA* fragments were generated by ligation of the 235 bp DNA fragment Plac-5′UTR with the 1847 bp DNA fragment SD^+^*gusA* and 1829 bp DNA fragment SD^-^*gusA* by fusion PCR, respectively. Plac-5′UTR was obtained by PCR amplification using the DNA of strain 8004 as template and the primer pair Plac-5′UTR-F/5′UTR-R (Table S2), and SD^+^*gusA* and SD^-^*gusA* were obtained by PCR amplification using the DNA of *E. coli* K12 as template and the primer pairs SD^+^-*gusA*-F/*gusA*-R and SD^-^-*gusA*-F/*gusA*-R (Table S2), respectively.

### Northern blotting and Western blotting

*Xcc* cells were cultured under specific growth conditions and collected at 24 h post-inoculation. Total RNA was isolated using the PureLink RNA Mini kit (Thermo Fisher Scientific, Shanghai, China), and 3–5 µg RNA was separated on 6 % denature (8 M urea) polyacrylamide gel and transferred to a positively charged nylon membrane (Roche Applied Science, Mannheim, Germany). After UV-crosslinking, the membrane was hybridized with a DIG-labelled RNA probe [prepared using a DIG RNA labelling kit (Roche Applied Science)] at 68 °C for 8 h. Signal bands were detected using the DIG-Northern Starter Kit (Roche Applied Science) and visualized with an ImageQuant LAS 500 imager (GE Healthcare, Beijing, China). Western blotting was performed as described previously [15].

### GUS activity assay and plant test

GUS activity was determined by measurement of the OD_415_ using ρ-nitrophenyl-*β*-d-glucuronide as a substrate, as described previously [18]. The virulence of *Xcc* strains was tested on the leaves of Chinese radish (*Raphanus sativus* L. var. radiculus Pers.) using the leaf-clipping method [15].

## Results

### *Xcc* encodes a *met* (*XC1251-XC1253*) operon that is essential for growth in the absence of methionine

Here, using the genome annotation of the *Xcc* strain 8004 [19] and the methionine biosynthetic pathway of *E. coli* [4], we constructed an *in silico* methionine biosynthetic pathway for *Xcc*. As shown in Fig. 1, all of the genes encoding the enzymes that accomplish the trans-sulfuration pathway from homoserine to methionine and the process from aspartate to homoserine are present in the genome of *Xcc* strain 8004. However, the gene (named *metY* in *E. coli*) encoding O-acetylhomoserine sulfhydrylase, which is indispensable for the direct sulfhydrylation pathway, is absent in the genome. This *in silico* model implies that *Xcc* synthesizes methionine *via* the trans-sulfuration pathway but not *via* a direct sulfhydrylation pathway. Notably, the genes encoding the enzymes converting aspartate semialdehyde to cystathionine, i.e. *XC1251* (*metA*), *XC1252* (*metB*) and *XC1253* (*hom*), are located together, while the genes encoding other enzymes involved in the biosynthesis of methionine from aspartate are scattered on the chromosome of *Xcc* strain 8004 (Fig. 1).

**Fig. 2.**
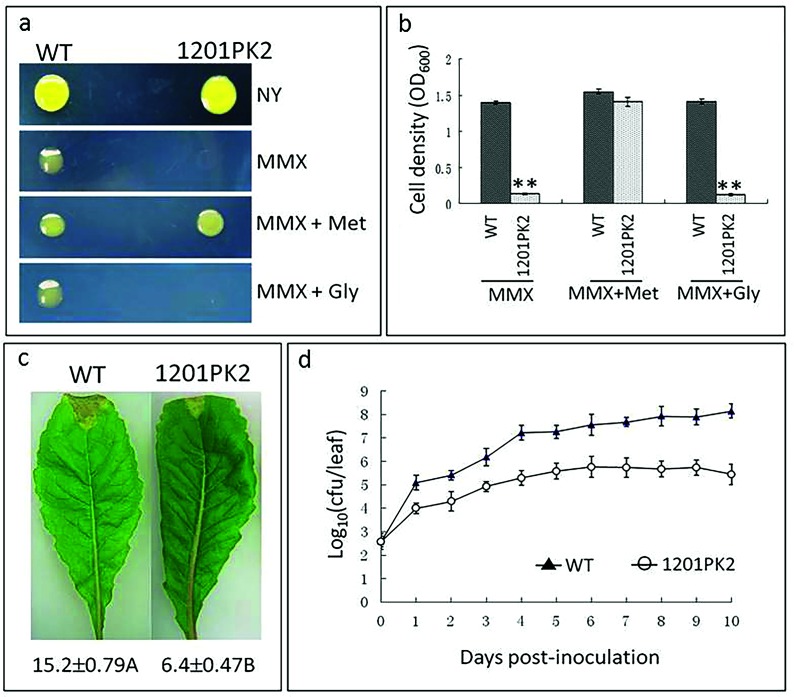
Inactivation of the *met* operon has an impact on *Xcc* virulence and growth in methionine-deficient medium and *in planta*. (a) and (b) Plate assay and quantitative liquid culture assay for detection of the growth of *Xcc* strains. NYG, nutrien- rich medium; MMX, minimal medium; MMX+Met or Gly, MMX supplemented with 1 mM of methionine or glycine. The photographs were taken at either 48 h (for NYG) or 72 h (for MMX and MMX+Met or Gly) post-incubation at 28 °C. The cell density was measured 72 h after incubation at 28 °C. (c) Black rot symptoms caused by *Xcc* strains on Chinese radish leaves. Images were taken at day 10 post-inoculation. Values under each leaf are the average lesion length (in mm) (mean±sd) from three repeats, each with 50 leaves. Different letters following the values indicate significant difference (*t*-test, *P*=0.01). (d) Growth rates of *Xcc* strains in inoculated leaves of Chinese radish. Bacterial cells were recovered from the inoculated leaves every day over a period of 10 days post-inoculation. Data are the mean±sd from a representative experiment, and similar results were obtained in two other independent experiments. WT, the wild-type strain 8004; 1201PK2, the *met* operon mutant strain.

Bioinformatic analysis of the ORFs *XC1251-XC1253* suggests that these genes are transcribed in the same direction and potentially co-transcribed as a single operon (Fig. 1). This was verified by Northern blotting analysis (Fig. 1). We named this the *met* (*XC1251-XC1253*) operon for simplification. To validate the involvement of the *met* operon in methionine biosynthesis, an insertion mutant strain named 1201PK2 (Table S1) was constructed. This mutant has an insertion in the first ORF (i.e. *XC1251*) of the operon, thus blocking the transcription of the whole operon. As illustrated in Fig. 2, the mutant strain grew well in the nutrient-rich medium (NYG) but was unable to grow in the nutrient-limited medium (MMX), while the wild-type strain 8004 grew well in both media. However, supplementing MMX with methionine restored the mutant’s ability to grow as well as the wild-type (Fig. 2). Interestingly, none of the other 19 standard amino acids, which were used as supplements under the same test conditions, could rescue the growth of the mutant in MMX. These results demonstrated that the mutant is methionine auxotroph and the *met* operon is essential for the growth of *Xcc* in the absence of methionine.

### The *met* operon is required for full virulence and *in planta* growth of *Xcc*

To investigate whether the *met* operon (and by inference methionine biosynthesis) is important for the pathogenicity of *Xcc*, we tested the virulence of the mutant strain 1201PK2 on the host plant Chinese radish by leaf-clipping assay [15]. The results showed that the disease symptoms caused by the mutant were significantly weaker compared to the wild-type. As shown in Fig. 2(c), at 10 days post-inoculation the wild-type produced disease symptoms with a mean lesion length of 15.2 mm, while the mean lesion length caused by the mutant was only 6.4 mm. This finding indicates that the *met* operon is required for full virulence of *Xcc*.

The mutant grew poorly in environments lacking methionine (Fig. 2a, b). For this reason, we further assessed the growth of the mutant *in planta*. A group of five inoculated leaves were homogenized and plated on NYG medium supplemented with appropriate antibiotics. Growth was recorded after incubation at 28 °C for 10 days. During the observation period, the number of mutant colonies recovered from the infected leaves was 10- to 100-fold lower than that of the wild-type (Fig. 2d). This finding demonstrates that the *met* operon (and by inference methionine biosynthesis) is important for *Xcc* to propagate regularly in host plants.

### *met* operon expression is controlled in a methionine-dependent manner

**Fig. 3.**
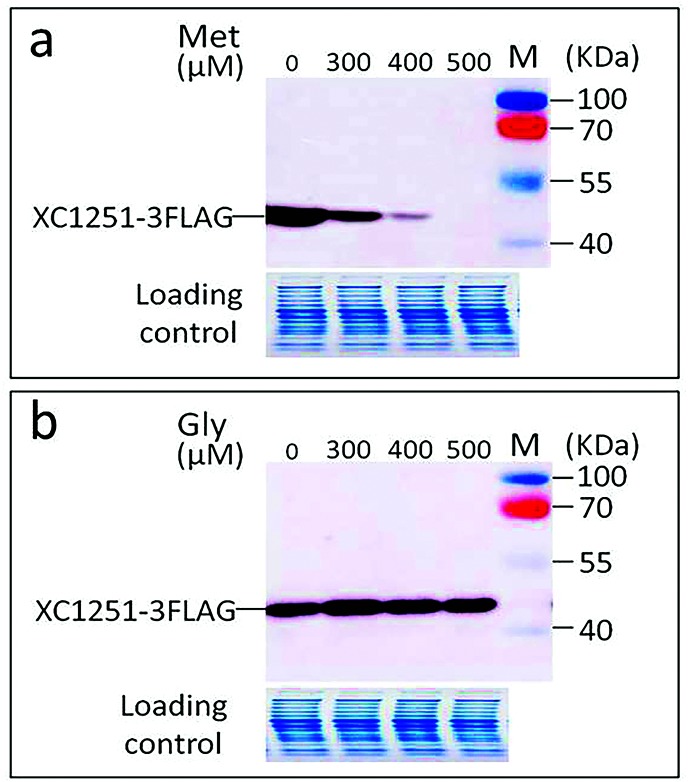
Western blotting detection of the effect of methionine on the expression of XC1251-3FLAG protein. Strain XC1251-3F was grown in minimal medium MMX or MMX supplemented with methionine (Met) (a) or glycine (Gly) (b) to a final concentration of 300, 400 and 500 µM, respectively. Cells were collected 24 h post-inoculation and total proteins were isolated from the cells. The level of XC1251-3FLAG protein in the total protein samples was detected by Western blotting analysis using anti-Flag monoclonal antibody as probe.

To gain an insight into how the expression of the *met* operon is controlled, a wild-type strain expressing a recombinant XC1251 protein (XC1251-3FLAG) was generated and named XC1251-3F (Table S1). This was achieved by adding 3×Flag tag to the C-terminus of XC1251 by in-frame fusing a DNA segment encoding 3×Flag tag to the 3′-end (just before the stop codon TGA) of *XC1251*, and introducing it into the genome of strain 8004 using a suicide vector-mediated unmarked allelic replacement method [17]. The expression level of the recombinant protein XC1251-3FLAG was assessed using Western blotting when the *Xcc* strain was grown in MMX with or without methionine. The result showed that XC1251-3FLAG protein expression was elevated in cells grown in MMX without methionine (Fig. 3a). However, the level of XC1251-3FLAG was reduced dramatically in the presence of methionine. No detectable XC1251-3FLAG protein was observed when the concentration of methionine was increased to 500 µM (Fig. 3a). Moreover, the addition of other standard amino acids (such as 500 µM glycine) did not effect XC1251-3FLAG expression (Fig. 3b), indicating that the repression of XC1251-3FLAG expression by methionine was specific. These results suggest that the *met* operon is expressed in a methionine-dependent manner, where activation and inactivation of the operon are dependent on the level of methionine in the surrounding environment.

### The 5′UTR, but not the promoter of the *met* operon, is involved in controlling expression in response to methionine availability

**Fig. 4.**
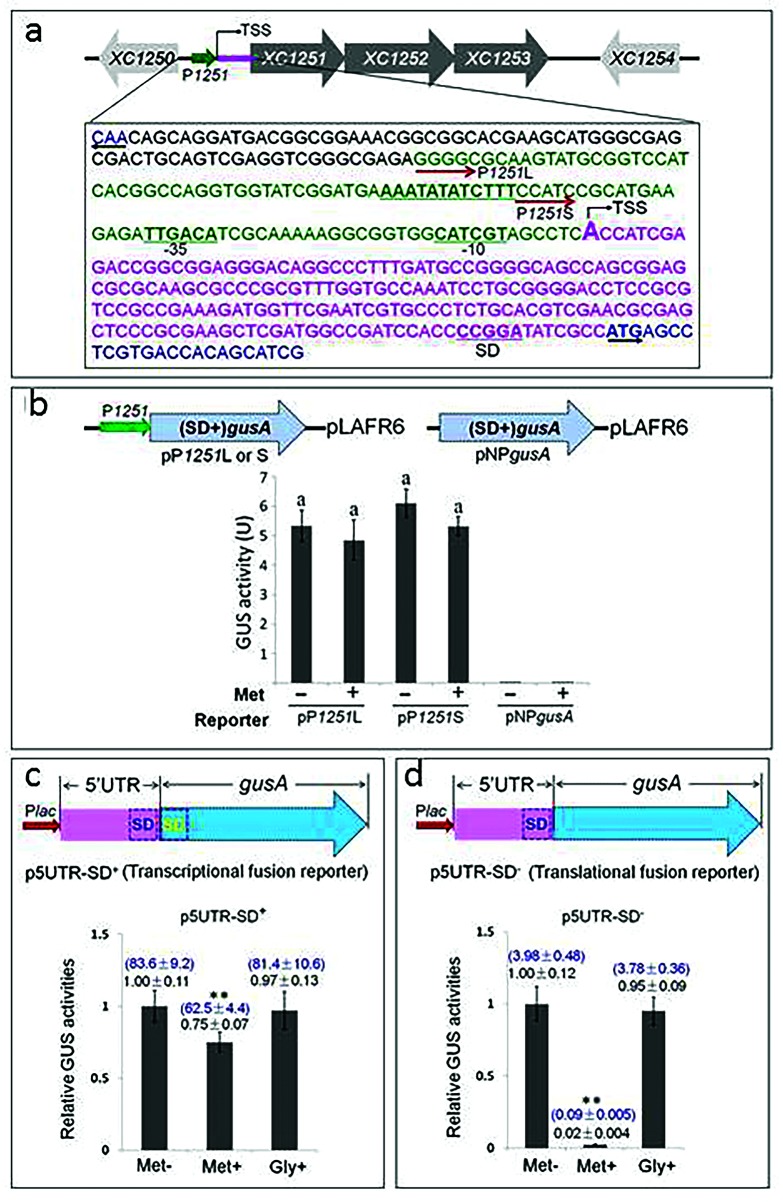
Characterization and functional analysis of the promoter region and the 5′UTR of the *met* operon. (a) Characterization of the promoter. The small green arrow represents the promoter (P_1251_) of the operon. TSS represents the identified transcriptional start site. The promoter-containing DNA sequences used for the construction of reporter plasmids are shown in green, while the 5’UTR sequence is in magenta. The −10/–35 region and the Shine-Dalgarno sequence (SD) are indicated. (b) Functional analysis of the promoter. The upper part shows the genetic organization of the reporter plasmids pP*1251*L and pP*1251*L. The lower part shows the GUS activities produced by the reporter strains 8004/pP*1251*L (pP*1251*L) and 8004/pP*1251*S (pP*1251*S), as well as the negative control strain 8004/pNP*gusA* (pNP*gusA*) grown for 72 h in MMX alone (Met−) and MMX with 1 mM methionine (Met+). Data are from a representative experiment, and similar results were obtained in two other independent experiments. The letters above the columns represent no significant difference at *P*=0.05 by *t*-test. (c) and (d) Functional analysis of the 5′UTR. The upper part shows the genetic organization of the 5′UTR-*gusA* transcriptional fusion reporter plasmid (p5UTR-SD^+^) and the translational fusion reporter plasmid (p5UTR-SD^−^). The lower part shows the GUS activities produced by the reporter strains 8004/p5′UTR-SD^+^ (p5UTR-SD^+^) and 8004/p5′UTR-SD^−^ (p5UTR-SD^−^) grown in MMX alone (Met−) and MMX with the addition of 1 mM methionine (Met+) or 1 mM glycine (Gly+). The asterisks above the columns represent significant difference at *P*<0.05 by *t*-test. The data in blue and black above the columns are the absolute and relative values, respectively. One unit of GUS activity was defined as milligrams (mg) of ρ-nitrophenol released from ρ-nitrophenyl *β*-d-glucuronide per minute per ml of bacterial culture (cell density: OD_600_=1.0). Data presented are from a representative experiment, and similar results were obtained in two other independent experiments.

The above findings suggest that *Xcc* may regulate the expression of the *met* operon by a feedback inhibition manner in response to methionine. To investigate whether feedback inhibition by methionine plays a role in controlling the transcriptional level of the *met* operon, we first determined the transcriptional start site (TSS) of the operon by 5’RACE (rapid amplification of cDNA ends) (Fig. S1). This analysis predicted putative −10 and −35 elements of the core promoter of the operon based on the identified TSS (Fig. 4a). Based on this, two promoter-*gusA* transcriptional fusion reporter plasmids, named pP*1251*L and pP*1251*S, were constructed by fusing 109 and 52 bp DNA sequences upstream of the TSS with the promoterless *gusA* gene (which still retained its Shine–Dalgano sequence) and cloning into the vector pLAFR6 (Fig. 4b). Subsequently, these reporter plasmids were introduced into strain 8004, generating reporter strains named 8004/pP*1251*L and 8004/pP*1251*S (Table S1). Simultaneously, a recombinant plasmid named pNP*gusA* (Table S1) was constructed, in which the promoterless *gusA* gene was cloned into the promoterless cloning sites of the vector pLAFR6 and introduced into strain 8004 as a negative control. GUS activity produced by the obtained reporter strains was determined after incubation for 72 h in MMX with or without methionine. The result demonstrated that the recombinant strains 8004/pP*1251*L and 8004/pP*1251*S produced similar GUS activities in MMX (Fig. 4b). This suggests that the 52 and 109 bp DNA segments upstream of the TSS of the operon have similar promoter activities. Contrary to expectations, both strains produced similar GUS activities in MMX with and without methionine supplementation (Fig. 4b). However, the control strain 8004/pNP*gusA* (Table S1) did not produce any detectable GUS activity (Fig. 4b). These data imply that the promoter of the *met* operon is not involved in the feedback regulation of methionine biosynthesis in response to methionine availability.

The fact that the promoter of the *met* operon did not respond to methionine availability suggests that the feedback control of this operon may be at the post-transcriptional level. Notably, the operon has a very long (190 nt) 5′UTR (Fig. 4a), implying that the 5′UTR may be responsible for feedback control. To test this possibility, 5′UTR-*gusA* transcriptional and translational fusion reporter plasmids, named p5′UTR-SD^+^ and p5′UTR-SD^-^, respectively, were constructed (Fig. 4c, d), where both fusions were driven by the *E. coli lac* promoter whose expression is constitutive in *Xcc*. The reporter plasmids were introduced into strain 8004, generating the reporter strains named 8004/p5′UTR-SD^+^ and 8004/p5′UTR-SD^−^ (Table S1). The GUS activities produced by the reporter strains grown in MMX with or without methionine were measured. The results showed that the GUS activity produced by the translational reporter was reduced by 98 % when methionine was added (Fig. 4d). Addition of glycine to the medium had no effect on the GUS activity produced by either of the reporters (Fig. 4c, d), indicating that the reduction in GUS activity by methionine was specific. These data denote that the 5’UTR plays a key role in the feedback regulation of the *met* operon, whose regulation appears to be mainly at the translational level. Interestingly, only 75 % GUS activity was produced by the transcriptional reporter when methionine was added (Fig. 4c), suggesting that the 5′UTR may also employ a transcription attenuation mechanism to regulate the *met* operon.

### The *met* operon is widely distributed among *Xanthomonas* species

**Fig. 5.**
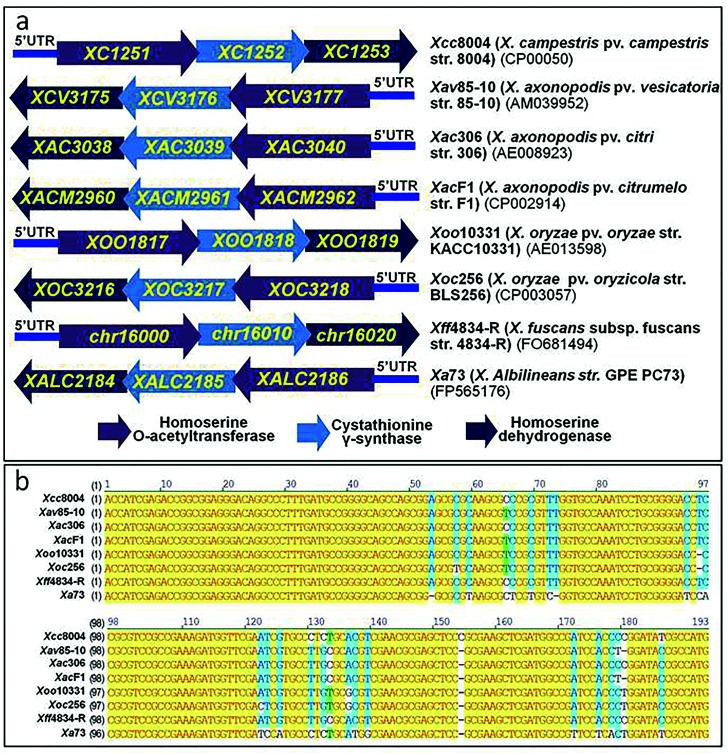
The met operon is highly conserved among sequenced *Xanthomonas* species. The arrows represent genes annotated in the genomes of *Xanthomonas* strains. The numbers in parentheses below the strain names are the access numbers in the NCBI database. The numbers above the arrows are the ORF names annotated.

A blast analysis revealed that the *met* operon containing *metA*, *metB* and *hom* genes is present in all completely sequenced *Xanthomonas* species, subspecies and pathovars (i.e. *X. campestris* pv. campestris, *X. axonopodis* pv. vesicatoria, *X. axonopodis* pv. citri, *X. axonopodis* pv. citrumelo, *X. oryzae* pv. oryzae, *X. oryzae* pv. oryzicola, *X. fuscans* subsp. fuscans and *X. albilineans*) (Fig. 5). On the contrary, the location of the *metA*, *metB* and *hom* genes is scattered among the genomes of other bacteria. Interestingly, a further blast search showed that the 5′UTR of the *met* operon is highly conserved among all sequenced *Xanthomonas* genomes, including those with a draft sequence present in the NCBI sequence database (https://blast.ncbi.nlm.nih.gov/) (Fig. 5, Table S3). In addition to *Xanthomoas* species, the highly conserved 5′UTR sequence is also found in *Pseudoxanthomonas suwonensis* and *Stenotrophomonas acidaminiphila* (Table S3).

## Discussion

To establish a successful infection, a plant-parasitic bacterium must be capable of utilizing plant compounds as nutrients for growth in addition to overcoming plant defences. However, the infected plant tissue is generally a nutrient-deficient environment where nutritional compounds such as nucleotides and amino acids are insufficient for bacterial growth. Thus, having the ability to synthesize nucleotides and amino acids from simpler compounds is critical for plant-parasitic pathogens to proliferate efficiently and invade hosts successfully. *Xcc* is an intercellular parasite that generally invades, colonizes and multiplies in the vascular tissues of cruciferous plants. In this work, we showed that an *Xcc* mutant strain defective for the *met* operon (and by inference methionine biosynthesis) caused a severe reduction in virulence and growth rate in the host plant. Given that the mutant grew as well as the wild-type in nutrient-rich medium, this result suggests that there was insufficient methionine in the vascular tissues of the host plant tested for *Xcc* growth, and that *de novo* synthesis of methionine is crucial for *Xcc* to proliferate and establish an infection in the host.

As illustrated in Fig. 1, *Xcc* possesses genes encoding a complete set of enzymes required for methionine synthesis from aspartate *via* the trans-sulfuration pathway. Certain bacteria have developed an alternative pathway, called the direct sulfhydrylation pathway, to synthesize methionine, in which O-acetylhomoserine is directly converted to homocysteine by O-acetylhomoserine sulfhydrylase, encoded by *metY* [3]. However, it seems that members of the *Xanthomonas* genus do not employ the direct sulfhydrylation pathway to synthesize methionine, as the *metY* gene is missing in the genomes of all sequenced *Xanthomonas* strains as revealed by a blast search against the NCBI sequence database (https://blast.ncbi.nlm.nih.gov/).

Previous studies reported that in Gram-negative bacteria the expression of the genes involved in methionine synthesis is regulated by a mechanism of transcriptional-level feedback inhibition [4]. In *E. coli* the *met* genes involved in methionine biosynthesis are regulated by the activator MetR and the repressor MetJ, which bind directly to the MetR- and MetJ-binding motifs present in the promoters of *met* genes [20]. The *Xcc* strain 8004 encodes a MetR homologue (*XC0327*) that shares 37 % identity with the *E. coli* MetR protein, although *metJ* is missing in its genome [19]. However, bioinformatics analysis revealed that there is no typical MetR-binding motif present in any of the promoters of the genes involved in methionine biosynthesis, as illustrated in Fig. 1. Given that the promoter of the *Xcc met* operon does not respond to methionine availability (Fig. 4b), the MetR homologue in *Xcc* may not have a direct effect on the expression of *met* genes. It was reported that *Ralstonia solanacearum* MetR regulates the expression of *metE* but not *metH* [13], both of which are regulated by MetR in *E. coli* and *Salmonella typhimurium* [21]. Interestingly, *R. solanacearum metE*, *metH* and *metR* mutants are not auxotrophic for methionine [13]. Whether the MetR homologue in *Xcc* plays a role in methionine biosynthesis needs to be further investigated.

As mentioned above, Gram-positive bacteria generally utilize RNA regulators such as the S-box and T-box riboswitches to control the expression of genes involved in *de novo* methionine biosynthesis at the post-transcriptional level. Based on the results of promoter-*gusA* assay and 5′UTR-*gusA* fusion reporter analysis, we conclude that the *Xcc met* operon is constitutively transcribed regardless of whether methionine is sufficient or deficient in the environment, but that translation initiation of the operon mRNA is tightly controlled by its 5′UTR in response to methionine availability. Consistently, the predicted secondary structure of the 5′UTR shows that the SD sequence of the first ORF XC1251 is blocked (Fig. S2), suggesting that the 5’UTR can regulate the expression of XC1251 at the translational level. To the best of our knowledge, this is the first work showing that the 5’UTR of the *met* operon mRNA in a Gram-negative bacterium plays a key role in the regulation of *de novo* methionine biosynthesis. It is known that methionine’s downstream product SAM can be recycled. During methylation, SAM is converted to S-adenosylhomocysteine (SAH), which is then hydrolysed by SAH hydrolase to form the upstream metabolite of methionine, homocysteine [2]. It was demonstrated that a riboswitch at the 5′UTR of the gene encoding SAH hydrolase senses SAH and activates genes involved in co-enzyme recycling in Gram-negative bacteria, such as *Pseudomonas syringae* [22]. The riboswitch is highly conserved in the 5′UTRs of the genes predicted to encode SAH hydrolase in *Xcc* strains, suggesting that *Xcc* may also use the 5′UTR of SAH hydrolase-encoding gene to regulate SAM recycling [22].

Interestingly, an Rfam search (http://rfam.xfam.org/) showed that the 1–120 nt region in the 190 nt 5′UTR of the *Xcc met* operon displays 52 % sequence identity with the aptamer domain of the *yitJ* SAM-I riboswitch of *Bacillus subtilis*. However, there is no such SAM-I riboswitch-like sequence present in the 5′UTR regions of other genes involved in *Xcc* methionine biosynthesis. This suggests that the expression of the *met* operon and other *met* genes is differentially regulated in *Xcc*. Given that the *met* operon and its 5′UTR are highly conserved in *Xanthomonas* genus, to further investigate the detailed molecular mechanism by which the 5′UTR controls the expression of the *met* operon in *Xcc* will be very valuable in regard to our understanding of methionine biosynthesis regulation in *Xanthomonas*.

## Supplementary Data

Supplementary File 1
