# Supplementary material for: The Gram-negative phytopathogen Xanthomonas campestris pv. campestris employs a 5'UTR as a feedback controller to regulate methionine biosynthesis
Source: Microbiology (Reading). 2018 Jul 19;164(9):1146–55. doi: 10.1099/mic.0.000690 (PMC6230763; doi:10.1099/mic.0.000690)
Supplement: Supplementary File 1 [file mic-164-1146-s001.pdf]

Supplemental Materials

Table S1. Bacterial strains and plasmids used in this work.

Table S2. Distribution of the 5'UTR of *met* operon in other bacterial genomes.

Table S3. Primer sets and DNA oligos used in this work.

Fig. S1. Identification of the transcription start site (TSS) of the *met* operon (*XC1251*-*XC1253*) by 5'-RACE.

Fig. S2. The potential secondary structure of the 5'UTR of the *Xcc met* operon.

Table S1. Bacterial strains and plasmids used in this work.

Strains and plasmids	Relevant characteristics*	Source or reference
E. coli strains		
K12	Wild-type *E. coli* strain	[1]
ED8767/pRK2073	RecA56, metB, hsdS, supE, supF, containing pRK2073, Spc^r	[2]
DH5α	F-recA1Φ80d LacZ ΔM15	[3]
Xcc strains		
8004	Wild-type strain, Rif^r	[4]
1201PK2	As 8004, but *XC1251* (*metA*)::pK18mob, Rif^r, Kan^r	This study
XC1251-3F	As 8004, but the 3'-end of *XC1251* coding region with a in frame insertion of 3×FLAG-coding sequence, Rif^r	This study
8004/pP*1251*L	8004 harboring pP*1251*L , Rif^r, Tc^r	This study
8004/pP*1251*S	8004 harboring pP*1251*S , Rif^r, Tc^r	This study
8004/pNP*gusA*	8004 harboring pNP*gusA*, Rif^r, Tc^r	This work
8004/p5UTR-SD^+	8004 harboring p5UTR-SD^+ , Rif^r, Tc^r	This study
8004/p5UTR-SD^-	8004 harboring p5UTR-SD^- , Rif^r, Tc^r	This study
Plasmids		
pLAFR6	Broad host range cloning vector, Tc^r	[5]
pRK2073	helper plasmid, Tra+, Mob+, ColE1, Spc^r	[6]
pK18mob	Suicide plasmid, Kan^r	[7]
pK18mobsacB	The suicide plasmid pK18mob containing a *sacB* gene, Kan^r	[7]
pK1251	pK18mob containing the 392-bp internal fragment of *XC1251*, Kan^r	This study
pKS1251-3F	pK18mobsacB containing the upstream and downstream flanking sequence of *XC1251* and	This study

	3×FLAG-coding sequence. Kan^r.	
pP*1251*L	pLAFR6 containing a DNA fragment in which P1251L was fused with the SD-containing *gusA*, Tc^r	This study
pP*1251*S	pLAFR6 containing a DNA fragment in which P1251S was fused with the SD-containing *gusA*, Tc^r	This study
pNP*gusA*	pLAFR6 containing the promoterless SD-containing *gusA*, Tc^r	This work
p5UTR-SD$^+$	pLAFR6 containing a DNA fragment in which the *E.coli lac* promoter and the 5'UTR of *XC1251-XC1253* operon of *Xcc* were linked together and fused with the SD-containing *gusA*, Tc^r	This study
p5UTR-SD$^-$	pLAFR6 containing a DNA fragment in which the *E.coli lac* promoter and the 5'UTR of *XC1251-XC1253* operon of *Xcc* were linked together and fused with the SD-less *gusA*, Tc^r	This study

*Rif^r, rifampicin-resistant; Kan^r, kanamycin-resistant; Spc^r, spectinomycin-resistant; Tc^r, tetracycline-resistant.

References

[1] Bachman B. Pedigrees of some mutant strains of *Escherichia coli* K12. *Bacteriological Rev* 1972;36:527-557.

[2] Murray NE, Brammar WJ, Murray K. Lambdoid phages that simplify the recovery of in vitro recombinants. *Mol Gen Genet* 1977;150:53-61.

[3] Hanahan D. Studies on transformation of *E. coli* with plasmids. *J Mol Biol* 1983;166:557-580.

[4] Daniels MJ, Barber CE, Turner PC, Sawczyc MK, Byrde RJ, Fielding AH. Cloning of genes involved in pathogenicity of *Xanthomonas campestris* pv. *campestris* using the broad host range cosmid pLAFR1. *EMBO J* 1984;3:3323-3328.

[5] Huynh TV, Dahlbeck D, Staskawicz BJ. Bacterial blight of soybean: regulation of a pathogen gene determining host cultivar specificity. *Science* 1989;245:1374-1377.

[6] Leong SA, Ditta GS, Helinski DR. Heme biosynthesis in *Rhizobium*. Identification of a cloned gene coding for delta-aminolevulinic acid synthetase from *Rhizobium meliloti*. *J Biol Chem* 1982;257:8724-8730.

[7] Schäfer A, Tauch A, Jäger W, Kalinowski J, Thierbach G, Pühler A. Small mobilizable multi-purpose cloning vectors derived from the *Escherichia coli* plasmids pK18 and pK19: selection of defined deletions in the chromosome of *Corynebacterium glutamicum*. *Gene* 1994;145:69-73.

Table S2 Primer sets and DNA oligos used in this work

Primer pairs and their purpose	Sequence (5’ to 3’)*	Product (bp)
For mutant construction		
1251M-F/1251M-R	CCCGAATTCGTGACCACAGCATCGCCAC/CCCAAGCTTAGCGCATCCAGCAGTGCGG	392
P18con-F	GCCGATTCATTAATGCAGCTGGCAC	
1251con-R	TCATGCGGTTTCTCCGGTC	
1251-3F-U-F/1251-3F-U-R	CCCGGATCCCGCGTCGCGCCGACTGTTGGCCTTCGATTTC/***CGGTGATTATAAAGATCATGATATCGACTACAAAGATGACGACGATAAA***TGCGGTTTCTCCGGTCGTG	781
1251-3F-D-F/1251-3F-D-R	***ATCTTTATAATCACCGTCATGGTCTTTGTAGTC***TGAGCTTTCGTGACCCTGCCGACGCGCC/CCCTCTAGAAGCGCCGGAGACAGGAAGGT	607
1251-3F-U-F/1251-3F-D-R	CCCGGATCCCGCGTCGCGCCGACTGTTGGCCTTCGATTTC/ CCCTCTAGAAGCGCCGGAGACAGGAAGGT	1388
For construction of promoter-*gusA* reporters		
P1251L-SDgusA-F/gusA-R	CCCGAATTCGGGGCGCAAGTATGCGGTCCATCACGGCCAGGTGGTATCGGATGAAAATATATCTTTCCATCCGCATGAAGAGATTGACATCGCAAAAAGGCGGTGGCATCGTAGCCTCATGAGGAGTCCCTTATGTTACGTCCTGTAGAAACC/CCCAAGCTTTCATTGTTTGCCTCCCTGCTGCGG	1935
P1251S-SDgusA-F/gusA-R	CCCGAATTCCCATCCGCATGAAGAGATTGACATCGCAAAAAGGCGGTGGCATCGTAGCCTCATGAGGAGTCCCTTATGTTACGTCCTGTAGAAACCCC/CCCAAGCTTTCATTGTTTGCCTCCCTGCTGCGG	1878
For construction of 5’UTR-*gusA* reporters		
Plac-5’UTR-F/5’UTR-R	CCCGAATTCGGCTTTACACTTTATGCTTCCGGCTCGTATGTTGTGTGGAACCATCGAGACCGGCGGAGGG/CATGGCGATATCCGGGGTGG	235
SD$^+$-*gusA*-F/*gusA*-R	CCCCGGATATCGCCATGATTGAGGAGTCCCTTATGTTACGTCCTGTAGAAACCCC/CCCAAGCTTTCATTGTTTGCCTCCCTGCTGCGG	1847
SD$^-$-*gusA*-F/*gusA*-R	CCCCGGATATCGCCATGTTACGTCCTGTAGAAACCCCAACCC/CCCAAGCTTTCATTGTTTGCCTCCCTGCTGCGG	1829

* Added restrict sites were underlined. 3×FLAG-coding sequences were shown in bold italic letters. *lac* promoter sequence was highlighted in green, and the SD sequence of *gusA* was highlighted in red.

Table S3. Distribution of the 5'UTR of *met* operon in other bacterial genomes

Genome (GenBank Sequence ID)	The 5'UTR sequence of *met* operon (Genome location)*	Identities (%)	The first downstream gene
Xanthomonas campestris pv. *campestris* strain ATCC 33913 (AE008922.1)	ACCATCGAGACCGGCGGAGGGACAGGCCC<br>TTTGATGCCGGGGCAGCCAGCGGAGCGCG<br>CAAGCGCCCGCGTTTGGTGCCAAATCCTGC<br>GGGGACCTCCGCGTCCGCCGAAAGATGGT<br>TCGAATCGTGCCCTCTGCACGTCGAACGCG<br>AGCTCCCGCGAAGCTCGATGGCCGATCCAC<br>CCCGGATATCGCCATG (3379770-3379578)	100	homoserine O-acetyltransferase (*metA*)
Xanthomonas campestris pv. *campestris* strain 17 (CP011946.1)	ACCATCGAGACCGGCGGAGGGACAGGCCC<br>TTTGATGCCGGGGCAGCCAGCGGAGCGCG<br>CAAGCGCCCGCGTTTGGTGCCAAATCCTGC<br>GGGGACCTCCGCGTCCGCCGAAAGATGGT<br>TCGAATCGTGCCCTCTGCACGTCGAACGCG<br>AGCTCCCGCGAAGCTCGATGGCCGATCCAC<br>CCCGGATATCGCCATG (3304680- 3304488)	100	homoserine O-acetyltransferase (*metA*)
Xanthomonas campestris pv. *campestris* strain ICMP 4013(CP012146.1)	ACCATCGAGACCGGCGGAGGGACAGGCCC<br>TTTGATGCCGGGGCAGCCAGCGGAGCGCG<br>CAAGCGCCCGCGTTTGGTGCCAAATCCTGC<br>GGGGACCTCCGCGTCCGCCGAAAGATGGT<br>TCGAATCGTGCCCTCTGCACGTCGAACGCG<br>AGCTCCCGCGAAGCTCGATGGCCGATCCAC<br>CCCGGATATCGCCATG (1506299-1506491)	100	homoserine O-acetyltransferase (*metA*)
Xanthomonas campestris pv. *raphani* 756C (CP002789.1)	ACCATCGAGACCGGCGGAGGGACAGGCCC<br>TTTGATGCCGGGGCAGCCAGCGGAGCGCG<br>CAAGCGCCCGCGTTTGGTGCCAAATCCTGC<br>GGGGACCTCCGCGTCCGCCGAAAGATGGT<br>TCGAATCGTGCCCTCTGCACGTCGAACGCG<br>AGCTCCCGCGAAGCTCGATGGCCGATCCAC<br>CCCGGATATCGCCATG (3408936-3408744)	100	homoserine O-acetyltransferase (*metA*)
Xanthomonas campestris pv. *campestris* strain B100 (AM920689.1)	ACCATCGAGACCGGCGGAGGGACAGGCCC<br>TTTGATGCCGGGGCAGCCAGCGGAGCGCG<br>CAAGCGCACGCGTTTGGTGCCAAATCCTGC<br>GGGGACCTCCGCGTCCGCCGAAAGATGGT<br>TCGAATCGTGCCCTCTGCACGTCGAACGCG<br>AGCTCCCGCGAAGCTCGATGGCCGATCCAC<br>CCCGGATATCGCCATG (1507888- 1508080)	99	homoserine O-acetyltransferase (*metA*)
Xanthomonas campestris strain 17(CP011256.1)	ACCATCGAGACCGGCGGAGGGACAGGCCC<br>TTTGATGCCGGGGCAGCCAGCGGAGCGCG<br>CAAGCGCCCGCGTTTGGTGCCAAATCCTGC	98	homoserine O-acetyltransferase (*metA*)

	GGGGACCTCCGCGTCCGCCGAAAGATGGT<br>TCGAATCGTGCCTTGCGCACGTCGAACGCG<br>AGCTCCCGCGAAGCTCGATGGCCGATCCAC<br>CCCGGATATCGCCATG (2314417-2314225)		
Xanthomonas arboricola pv. *juglandis* strain Xaj 417(CP012251.1)	ACCATCGAGACCGGCGGAGGGACAGGCCC<br>TTTGATGCCGGGGCAGCCAGCGGAGCGCG<br>CAAGCGCCCGCGTTTGGTGCCAAATCCTGC<br>GGGGATCTCCGCGTCCGCCGAAAGATGGTT<br>CGAATCGTGCCTTGCGCACGTCGAACGCGA<br>GCTCCCGCGAAGCTCGATGGCCGATCCACC<br>CCGGATATCGCCATG (2993387- 2993195)	98	homoserine O-acetyltransferase (*metA*)
Xanthomonas gardneri strain ICMP 7383 (CP018731.1)	ACCATCGAGACCGGCGGAGGGACAGGCCC<br>TTTGATGCCGGGGCAGCCAGCGGAGCGCG<br>CAAGCGCCCGCGTTTGGTGCCAAATCCTGC<br>GGGGACCTGCGCGTCCGCCGAAAGATGGT<br>TCGATTTGTGCCTTGTGCACGTCGAACGCG<br>AGCTCCCGCGAAGCTCGATGGCCGATCCAC<br>CCCGGATATCGCCATG (3711209- 3711017)	97	homoserine O-acetyltransferase (*metA*)
Xanthomonas vesicatoria ATCC 35937 strain LMG 911(CP018725.1)	ACCATCGAGACCGGCGGAGGGACAGGCCC<br>TTTGATGCCGGGGCAGCCAGCGGAGCACG<br>CAAGTGCCCGCGTTTGGTGCCAAATCCTGC<br>GGGGACCTCCGCGTCCGCCGAAAGATGGT<br>TCGAATCGTGCCTTGCGCACGTCGAACGCG<br>AGCTCCCGCGAAGCTCGATGGCCGATCCAC<br>CCCGGATATCGCCATG (1368927- 1368735)	97	homoserine O-acetyltransferase (*metA*)
Xanthomonas vesicatoria strain LM159 (CP018470.1)	ACCATCGAGACCGGCGGAGGGACAGGCCC<br>TTTGATGCCGGGGCAGCCAGCGGAGCACG<br>CAAGTGCCCGCGTTTGGTGCCAAATCCTGC<br>GGGGACCTCCGCGTCCGCCGAAAGATGGT<br>TCGAATCGTGCCTTGCGCACGTCGAACGCG<br>AGCTCCCGCGAAGCTCGATGGCCGATCCAC<br>CCCGGATATCGCCATG (1672837-1672645)	97	homoserine O-acetyltransferase (*metA*)
Xanthomonas gardneri strain JS749-3 (CP018728.1)	ACCATCGAGACCGGCGGAGGGACAGGCCC<br>TTTGATGCCGGGGCAGCCAGCGGAGTGCG<br>CAAGCGCCCGCGTTTGGTGCCAAATCCTGC<br>GGGGACCTCCGCGTCCGCCGAAAGATGGT<br>TCGAATCGTGCCTTGTGCACGTCGAACGCG<br>AGCTCC-GCGAAGCTCGATGGCCGATCCAC<br>CCCGGATATCGCCATG (4131928-4131737)	97	homoserine O-acetyltransferase (*metA*)
Xanthomonas citri pv. *citri* strain LH2019 (CP018858.1)	ACCATCGAGACCGGCGGAGGGACAGGCCC<br>TTTGATGCCGGGGCAGCCAGCGGAGCGCG<br>CAAGCGCCCGCGTTTGGTGCCAAATCCTGC	96	homoserine O-acetyltransferase (*metA*)

	GGGGACCTCCGCGTCCGCCGAAAGATGGT<br>TCGAATCGTGCCTTGCGCACGTCGAACGCG<br>AGCT-CCGCGAAGCTCGATGGCCGATCCAC<br>CCCGGATACCGCCATG (84335- 84526)		
Xanthomonas citri pv. *citri* strain LH276 (CP018854.1)	ACCATCGAGACCGGCGGAGGGACAGGCCC<br>TTTGATGCCGGGGCAGCCAGCGGAGCGCG<br>CAAGCGCCCGCGTTTGGTGCCAAATCCTGC<br>GGGGACCTCCGCGTCCGCCGAAAGATGGT<br>TCGAATCGTGCCTTGCGCACGTCGAACGCG<br>AGCT-CCGCGAAGCTCGATGGCCGATCCAC<br>CCCGGATACCGCCATG (3881185- 3880994)	96	homoserine O-acetyltransferase (*metA*)
Xanthomonas citri pv. *citri* strain LJ207-7 (CP018850.1)	ACCATCGAGACCGGCGGAGGGACAGGCCC<br>TTTGATGCCGGGGCAGCCAGCGGAGCGCG<br>CAAGCGCCCGCGTTTGGTGCCAAATCCTGC<br>GGGGACCTCCGCGTCCGCCGAAAGATGGT<br>TCGAATCGTGCCTTGCGCACGTCGAACGCG<br>AGCT-CCGCGAAGCTCGATGGCCGATCCAC<br>CCCGGATACCGCCATG (1390894-1390703)	96	homoserine O-acetyltransferase (*metA*)
Xanthomonas citri pv. *citri* strain LL074-4 (CP018847.1)	ACCATCGAGACCGGCGGAGGGACAGGCCC<br>TTTGATGCCGGGGCAGCCAGCGGAGCGCG<br>CAAGCGCCCGCGTTTGGTGCCAAATCCTGC<br>GGGGACCTCCGCGTCCGCCGAAAGATGGT<br>TCGAATCGTGCCTTGCGCACGTCGAACGCG<br>AGCT-CCGCGAAGCTCGATGGCCGATCCAC<br>CCCGGATACCGCCATG (1945092- 1945283)	96	homoserine O-acetyltransferase (*metA*)
Xanthomonas axonopodis pv. *glycines* strain 8ra (CP017188.1)	ACCATCGAGACCGGCGGAGGGACAGGCCC<br>TTTGATGCCGGGGCAGCCAGCGGAGCGCG<br>CAAGCGCCCGCGTTTGGTGCCAAATCCTGC<br>GGGGACCTCCGCGTCCGCCGAAAGATGGT<br>TCGAATCGTGCCTTGCGCACGTCGAACGCG<br>AGCT-CCGCGAAGCTCGATGGCCGATCCAC<br>CCCGGATACCGCCATG (2082200-2082391)	96	homoserine O-acetyltransferase (*metA*)
Xanthomonas citri pv. *citri* strain jx-6 (CP011827.2)	ACCATCGAGACCGGCGGAGGGACAGGCCC<br>TTTGATGCCGGGGCAGCCAGCGGAGCGCG<br>CAAGCGCCCGCGTTTGGTGCCAAATCCTGC<br>GGGGACCTCCGCGTCCGCCGAAAGATGGT<br>TCGAATCGTGCCTTGCGCACGTCGAACGCG<br>AGCT-CCGCGAAGCTCGATGGCCGATCCAC<br>CCCGGATACCGCCATG (3534610-3534419)	96	homoserine O-acetyltransferase (*metA*)
Xanthomonas citri subsp. *citri* strain UI7 (CP008989.1)	ACCATCGAGACCGGCGGAGGGACAGGCCC<br>TTTGATGCCGGGGCAGCCAGCGGAGCGCG<br>CAAGCGCCCGCGTTTGGTGCCAAATCCTGC	96	homoserine O-acetyltransferase (*metA*)

	GGGGACCTCCGCGTCCGCCGAAAGATGGT<br>TCGAATCGTGCCTTGCGCACGTCGAACGCG<br>AGCT-CCGCGAAGCTCGATGGCCGATCCAC<br>CCCGGATACCGCCATG (3533684-3533493)		
Xanthomonas citri subsp. *citri* strain NT17 (CP008995.1)	ACCATCGAGACCGGCGGAGGGACAGGCCC<br>TTTGATGCCGGGGCAGCCAGCGGAGCGCG<br>CAAGCGCCCGCGTTTGGTGCCAAATCCTGC<br>GGGGACCTCCGCGTCCGCCGAAAGATGGT<br>TCGAATCGTGCCTTGCGCACGTCGAACGCG<br>AGCT-CCGCGAAGCTCGATGGCCGATCCAC<br>CCCGGATACCGCCATG (3558020-3557829)	96	homoserine O-acetyltransferase (*metA*)
Xanthomonas citri subsp. *citri* A306 (CP006857.1)	ACCATCGAGACCGGCGGAGGGACAGGCCC<br>TTTGATGCCGGGGCAGCCAGCGGAGCGCG<br>CAAGCGCCCGCGTTTGGTGCCAAATCCTGC<br>GGGGACCTCCGCGTCCGCCGAAAGATGGT<br>TCGAATCGTGCCTTGCGCACGTCGAACGCG<br>AGCT-CCGCGAAGCTCGATGGCCGATCCAC<br>CCCGGATACCGCCATG (3558013-3557822)	96	homoserine O-acetyltransferase (*metA*)
Xanthomonas fuscans subsp. *fuscans* strain 4834-R (FO681494.1)	ACCATCGAGACCGGCGGAGGGACAGGCCC<br>TTTGATGCCGGGGCAGCCAGCGGAGCGCG<br>CAAGCGCCCGCGTTTGGTGCCAAATCCTGC<br>GGGGACCTCCGCGTCCGCCGAAAGATGGT<br>TCGAATCGTGCCTTGCGCACGTCGAACGCG<br>AGCT-CCGCGAAGCTCGATGGCCGATCCAC<br>CCCGGATACCGCCATG (1847028-1847219)	96	homoserine O-acetyltransferase (*metA*)
Xanthomonas axonopodis pv. *citri* strain 306 (AE008923.1)	ACCATCGAGACCGGCGGAGGGACAGGCCC<br>TTTGATGCCGGGGCAGCCAGCGGAGCGCG<br>CAAGCGCCCGCGTTTGGTGCCAAATCCTGC<br>GGGGACCTCCGCGTCCGCCGAAAGATGGT<br>TCGAATCGTGCCTTGCGCACGTCGAACGCG<br>AGCT-CCGCGAAGCTCGATGGCCGATCCAC<br>CCCGGATACCGCCATG (3558019-3557828)	96	homoserine O-acetyltransferase (*metA*)
Xanthomonas perforans strain LH3 (CP018475.1)	ACCATCGAGACCGGCGGAGGGACAGGCCC<br>TTTGATGCCGGGGCAGCCAGCGGAGCGCG<br>CAAGCGTCCGCGTTTGGTGCCAAATCCTGC<br>GGGGACCTCCGCGTCCGCCGAAAGATGGT<br>TCGAATCGTGCCTTGCGCACGTCGAACGCG<br>AGCT-CCGCGAAGCTCGATGGCCGATCCAC<br>CC-GGATACCGCCATG (4938509- 4938699)	96	homoserine O-acetyltransferase (*metA*)
Xanthomonas euvesicatoria strain LMG930 (CP018467.1)	ACCATCGAGACCGGCGGAGGGACAGGCCC<br>TTTGATGCCGGGGCAGCCAGCGGAGCGCG<br>CAAGCGTCCGCGTTTGGTGCCAAATCCTGC	96	homoserine O-acetyltransferase (*metA*)

	GGGGACCTCCGCGTCCGCCGAAAGATGGT<br>TCGAATCGTGCCTTGCGCACGTCGAACGCG<br>AGCT-CCGCGAAGCTCGATGGCCGATCCAC<br>CC-GGATACCGCCATG (1061356- 1061546)		
Xanthomonas campestris pv. *vesicatoria* str. 85-10 (CP017190.1)	ACCATCGAGACCGGCGGAGGGACAGGCCC<br>TTTGATGCCGGGGCAGCCAGCGGAGCGCG<br>CAAGCGTCCGCGTTTGGTGCCAAATCCTGC<br>GGGGACCTCCGCGTCCGCCGAAAGATGGT<br>TCGAATCGTGCCTTGCGCACGTCGAACGCG<br>AGCT-CCGCGAAGCTCGATGGCCGATCCAC<br>CT-GGATACCGCCATG (1955038- 1954848)	96	homoserine O-acetyltransferase (*metA*)
Xanthomonas citri pv. *malvacearum* strain MSCT (CP017020.1)	ACCATCGAGACCGGCGGAGGGACAGGCCC<br>TTTGATGCCGGGGCAGCCAGCGGAGCGCG<br>CAAGCGTCCGCGTTTGGTGCCAAATCCTGC<br>GGGGACCTGCGCGTCCGCCGAAAGATGGT<br>TCGAATCGTGCCTTGCGCACGTCGAACGCG<br>AGCT-CCGCGAAGCTCGATGGCCGATCCAC<br>CCTGGACATCGCCATG (1507011- 1507202)	96	homoserine O-acetyltransferase (*metA*)
Xanthomonas fragariae isolate Fap29 (CP016833.1)	ACCATCGAGACCGGCGGAGGGACAGGCCC<br>TTTGATGCCGGGGCAGCCAGCGGAGCGCG<br>CAAGCGCCCGCGTTTGGTGCCAAATCCTGC<br>GGGGACTTT-GCGTCCGCCGAAAGATGGTT<br>CGAGTCGTGCCCTGGGCACGTCGAACGCG<br>AGCTCCCGCGAAGCTCGATGGCCGATCCAC<br>CCTGGACATCGCCATG (1274693- 1274884)	96	homoserine O-acetyltransferase (*metA*)
Xanthomonas fragariae isolate Fap21 (CP016830.1)	ACCATCGAGACCGGCGGAGGGACAGGCCC<br>TTTGATGCCGGGGCAGCCAGCGGAGCGCG<br>CAAGCGCCCGCGTTTGGTGCCAAATCCTGC<br>GGGGACTTT-GCGTCCGCCGAAAGATGGTT<br>CGAGTCGTGCCCTGGGCACGTCGAACGCG<br>AGCTCCCGCGAAGCTCGATGGCCGATCCAC<br>CCTGGACATCGCCATG (1274693- 1274884)	96	homoserine O-acetyltransferase (*metA*)
Xanthomonas axonopodis pv. *citrumelo* F1 (CP002914.1)	ACCATCGAGACCGGCGGAGGGACAGGCCC<br>TTTGATGCCGGGGCAGCCAGCGGAGCGCG<br>CAAGCGTCCGCGTTTGGTGCCAAATCCTGC<br>GGGGACCTCCGCGTCCGCCGAAAGATGGT<br>TCGAATCGTGCCTTGCGCACGTCGAACGCG<br>AGCTCC-GCGAAGCTCGATGGCCGATCCAC<br>CT-GGATACCGCCATG (3404872- 3404682)	96	homoserine O-acetyltransferase (*metA*)
Xanthomonas campestris pv. vesicatoria	ACCATCGAGACCGGCGGAGGGACAGGCCC<br>TTTGATGCCGGGGCAGCCAGCGGAGCGCG<br>CAAGCGTCCGCGTTTGGTGCCAAATCCTGC	96	homoserine O-acetyltransferase (*metA*)

(AM039952.1)	GGGGACCTCCGCGTCCGCCGAAAGATGGT<br>TCGAATCGTGCCTTGCGCACGTCGAACGCG<br>AGCTCC-GCGAAGCTCGATGGCCGATCCAC<br>CT-GGATACCGCCATG (3610521- 3610331)		
Xanthomonas oryzae pv. *oryzae* strain PXO602 (CP013679.1)	ACCATCGAGACCGGCGGAGGGACAGGCCC<br>TTTGATGCCGGGGCAGCCAGCGGAGCGCG<br>CAAGCGTCCGCGTTTGGTGCCAAATCCTGC<br>GGGGACC-CCGCGTCCGCCGAAAGATGGTT<br>CGACTCGTGCCTTGTGCGCGTCGAACGCGA<br>GCTCC-GCGAAGCTCGATGGCCGATCCACC<br>CTGGATACCGCCATG (1768597- 1768407)	95	homoserine O-acetyltransferase (*metA*)
Xanthomonas oryzae pv. *oryzae* strain PXO563 (CP013678.1)	ACCATCGAGACCGGCGGAGGGACAGGCCC<br>TTTGATGCCGGGGCAGCCAGCGGAGCGCG<br>CAAGCGTCCGCGTTTGGTGCCAAATCCTGC<br>GGGGACC-CCGCGTCCGCCGAAAGATGGTT<br>CGAATCGTGCCTTGTGCGCGTCGAACGCGA<br>GCTCC-GCGAAGCTCGATGGCCGATCCACC<br>CTGGATACCGCCATG (1849215- 1849405)	95	homoserine O-acetyltransferase (*metA*)
Xanthomonas oryzae pv. *oryzae* strain AXO1947 (CP013666.1)	ACCATCGAGACCGGCGGAGGGACAGGCCC<br>TTTGATGCCGGGGCAGCCAGCGGAGCGCG<br>CAAGCGTCCGCGTTTGGTGCCAAATCCTGC<br>GGGGACC-CCGCGTCCGCCGAAAGATGGTT<br>CGACTCGTGCCTTGTGCGCGTCGAACGCGA<br>GCTCC-GCGAAGCTCGATGGCCTATCCACC<br>CTGGATACCGCCATG (1491779- 1491969)	95	homoserine O-acetyltransferase (*metA*)
Xanthomonas oryzae pv. *oryzae* PXO99A (CP000967.2)	ACCATCGAGACCGGCGGAGGGACAGGCCC<br>TTTGATGCCGGGGCAGCCAGCGGAGCGCG<br>CAAGCGTCCGCGTTTGGTGCCAAATCCTGC<br>GGGGACC-CCGCGTCCGCCGAAAGATGGTT<br>CGAATCGTGCCTTGTGCGCGTCGAACGCGA<br>GCTCC-GCGAAGCTCGATGGCCGATCCACC<br>CTGGATACCGCCATG (3211162- 3210972)	95	homoserine O-acetyltransferase (*metA*)
Xanthomonas oryzae pv. *oryzae* MAFF 311018 (AP008229.1)	ACCATCGAGACCGGCGGAGGGACAGGCCC<br>TTTGATGCCGGGGCAGCCAGCGGAGCGCG<br>CAAGCGTCCGCGTTTGGTGCCAAATCCTGC<br>GGGGACC-CCGCGTCCGCCGAAAGATGGTT<br>CGAATCGTGCCTTGTGCGCGTCGAACGCGA<br>GCTCC-GCGAAGCTCGATGGCCGATCCACC<br>CTGGATACCGCCATG (1890533-1890723)	95	homoserine O-acetyltransferase (*metA*)
Xanthomonas oryzae pv. *oryzae* KACC 10331 (AE013598.1)	ACCATCGAGACCGGCGGAGGGACAGGCCC<br>TTTGATGCCGGGGCAGCCAGCGGAGCGCG<br>CAAGCGTCCGCGTTTGGTGCCAAATCCTGC	95	homoserine O-acetyltransferase (*metA*)

	GGGGACC-CCGCGTCCGCCGAAAGATGGTT CGAATCGTGCCTTGTGCGCGTCGAACGCGA GCTCC-GCGAAGCTCGATGGCCGATCCACC CTGGATACCGCCATG (1911400-1911590)		
Xanthomonas axonopodis pv. *dieffenbachiae* LMG 695 (CP014347.1)	ACCATCGAGACCGGCGGAGGGACAGGCCC TTTGATGCCGGGGCAGCCAGCGGAACGCT GCAAGCGTCCGCGTTTGGTGCCAAATCCTG CGGGGACCTCCGCGTCCGCCGAAAGATGG TTCGAATCGTGCCTTGCGCACGTCGAACGC GAGCT-CCGCGAAGCTCGATGGCCGATCCA CCCCGGATACCGCCATG (2412121-2412313)	95	homoserine O-acetyltransferase (*metA*)
Xanthomonas oryzae pv. *oryzicola* strain CFBP2286 (CP011962.1)	ACCATCGAGACCGGCGGAGGGACAGGCCC TTTGATGCCGGGGCAGCCAGCGGAGCGTG CAAGCGTCCGCGTTTGGTGCCAAATCCTGC GGGGACC-CCGCGTCCGCCGAAAGATGGTT CGACTCGTGCCTTGTGCGCGTCGAACGCGA GCTCC-GCGAAGCTCGATGGCCGATCCACC CTGGATACCGCCATG (3371623-3371433)	95	homoserine O-acetyltransferase (*metA*)
Xanthomonas oryzae pv. *oryzicola* strain RS105 (CP011961.1)	ACCATCGAGACCGGCGGAGGGACAGGCCC TTTGATGCCGGGGCAGCCAGCGGAGCGTG CAAGCGTCCGCGTTTGGTGCCAAATCCTGC GGGGACC-CCGCGTCCGCCGAAAGATGGTT CGACTCGTGCCTTGTGCGCGTCGAACGCGA GCTCC-GCGAAGCTCGATGGCCGATCCACC CTGGATACCGCCATG (3336310-3336120)	95	homoserine O-acetyltransferase (*metA*)
Xanthomonas oryzae pv. *oryzicola* strain L8 (CP011960.1)	ACCATCGAGACCGGCGGAGGGACAGGCCC TTTGATGCCGGGGCAGCCAGCGGAGCGTG CAAGCGTCCGCGTTTGGTGCCAAATCCTGC GGGGACC-CCGCGTCCGCCGAAAGATGGTT CGACTCGTGCCTTGTGCGCGTCGAACGCGA GCTCC-GCGAAGCTCGATGGCCGATCCACC CTGGATACCGCCATG (3351781-3351591)	95	homoserine O-acetyltransferase (*metA*)
Xanthomonas oryzae pv. *oryzicola* strain CFBP7331 (CP011958.1)	ACCATCGAGACCGGCGGAGGGACAGGCCC TTTGATGCCGGGGCAGCCAGCGGAGCGTG CAAGCGTCCGCGTTTGGTGCCAAATCCTGC GGGGACC-CCGCGTCCGCCGAAAGATGGTT CGACTCGTGCCTTGTGCGCGTCGAACGCGA GCTCC-GCGAAGCTCGATGGCCGATCCACC CTGGATACCGCCATG (1643852-1644042)	95	homoserine O-acetyltransferase (*metA*)
Xanthomonas oryzae pv. *oryzicola* strain BLS279 (CP011956.1)	ACCATCGAGACCGGCGGAGGGACAGGCCC TTTGATGCCGGGGCAGCCAGCGGAGCGTG CAAGCGTCCGCGTTTGGTGCCAAATCCTGC	95	homoserine O-acetyltransferase (*metA*)

	GGGGACC-CCGCGTCCGCCGAAAGATGGTT<br>CGACTCGTGCCTTGTGCGCGTCGAACGCGA<br>GCTCC-GCGAAGCTCGATGGCCGATCCACC<br>CTGGATACCGCCATG (3296444-3296254)		
Xanthomonas oryzae pv. *oryzicola* BLS256 (CP003057.2)	ACCATCGAGACCGGCGGAGGGACAGGCCC<br>TTTGATGCCGGGGCAGCCAGCGGAGCGTG<br>CAAGCGTCCGCGTTTGGTGCCAAATCCTGC<br>GGGGACC-CCGCGTCCGCCGAAAGATGGTT<br>CGACTCGTGCCTTGTGCGCGTCGAACGCGA<br>GCTCC-GCGAAGCTCGATGGCCGATCCACC<br>CTGGATACCGCCATG (3290526-3290336)	95	homoserine O-acetyltransferase (*metA*)
Xanthomonas translucens pv. *translucens* DSM 18974 isolate peng1 (LT604072.1)	ACCATCGAGACCGGCGGAGGGACAGGCCC<br>TTTGATGCCGGGGCAGCCAGCGG-GCGCGC<br>AAGCGACCGTGTA-GGTGCCAAATCCTGCG<br>GGGACCACCGCGTCCGCCGAAAGATGGTT<br>CGATTCGTGCCTTCCGCACGTCGAACGCGA<br>GCT-CCGCGAAGCTCGATGGCCGTTCCTCA<br>CCGGATACCGCCATG (1357175-1357364)	93	homoserine O-acetyltransferase (*metA*)
Xanthomonas translucens pv. *undulosa* strain Xtu 4699 (CP008714.1)	ACCATCGAGACCGGCGGAGGGACAGGCCC<br>TTTGATGCCGGGGCAGCCAGCGG-GCGCGC<br>AAGCGACCGTGTA-GGTGCCAAATCCTGCG<br>GGGACCACCGCGTCCGCCGAAAGATGGTT<br>CGATTCGTGCCTTCCGCACGTCGAACGCGA<br>GCT-CCGCGAAGCTCGATGGCCGTTCCTCA<br>CCGGATACCGCCATG (3086816-3086627)	93	homoserine O-acetyltransferase (*metA*)
Xanthomonas sacchari strain R1 (CP010409.1)	ACCATCGAGACCGGCGGAGGGACAGGCCC<br>TTTGATGCCGGGGCAGCCAGCGGA-CGCGC<br>AAGCGACCGTGTA-GGTGCCAAATCCTGCG<br>GGGACCACCGCGTCCGCCGAAAGATGGTT<br>CGATCCATGCCCTCTGCATGGCGAACGCGA<br>GCT-CCGCGAAGCTCGATGGCCGTTCCTCTC<br>CGGATACCGCCATG (237680-237869)	92	homoserine O-acetyltransferase (*metA*)
Xanthomonas albilineans GPE PC73 (FP565176.1)	ACCATCGAGACCGGCGGAGGGACAGGCCC<br>TTTGATGCCGGGGCAGCCAGCGG-GCGCGT<br>AAGCGCTCGTGTC-GGTGCCAAATCCTGCG<br>GGGATCCACGCGTCCGCCGAAAGATGGTT<br>CGATCCATGCCCTCTGCATGGCGAACGCGA<br>GCTCC-GCGAAGCTCGATGGCCGTTCCTCA<br>CTGGATATCGCCATG (2581129- 2580948)	90	homoserine O-acetyltransferase (*metA*)
Pseudoxanthomonas suwonensis strain J1 (CP011144.1)	ACCATCGAGACCGGCGGAGGGACAGGCCC<br>TTTGATGCCGGGGCAGCCAGCGGA-CGCGC<br>AAGCGGCCGTGTA-GGTGCCAAATCCTGCG	89	homoserine O-acetyltransferase (*metA*)

	GGGACCTTCGCGTCCGCCGAAAGATGGTTC GAACCGTGCTCTCCGCACGGCGAACGCGG GCTCCCGAAAGAGCTCGATGGC-GAGTTCC CTCCGGACATCGCCATG (2938209-2938399)		
Stenotrophomonas acidaminiphila strain ZAC14D2_NAIMI4_2 (CP012900.1)	ACCATCGAGACCGGCGGAGGGACAGGCCC TTTGATGCCGGGGCAACCAGCAG-GTGCGC AAGCGCCTGTGCCTGGTGCCAAATCCTGCG GGGACCACCGCGTCCGCCGAAAGATGGTT CGATCCGTGCC--CTGCACGGCGAACACGG GCTCCCGCGAAGCTCGATGGCCGATCCTTA CCGGATATCGCCATG (2922649-2922462)	88	homoserine O-acetyltransferase (*metA*)

*Red letters indicate the nucleotides that are different from the 5'UTR at the same positions. - indicates the nucleotide is missing there. The start codon ATG of the first downstream gene *metA* is indicated in green.

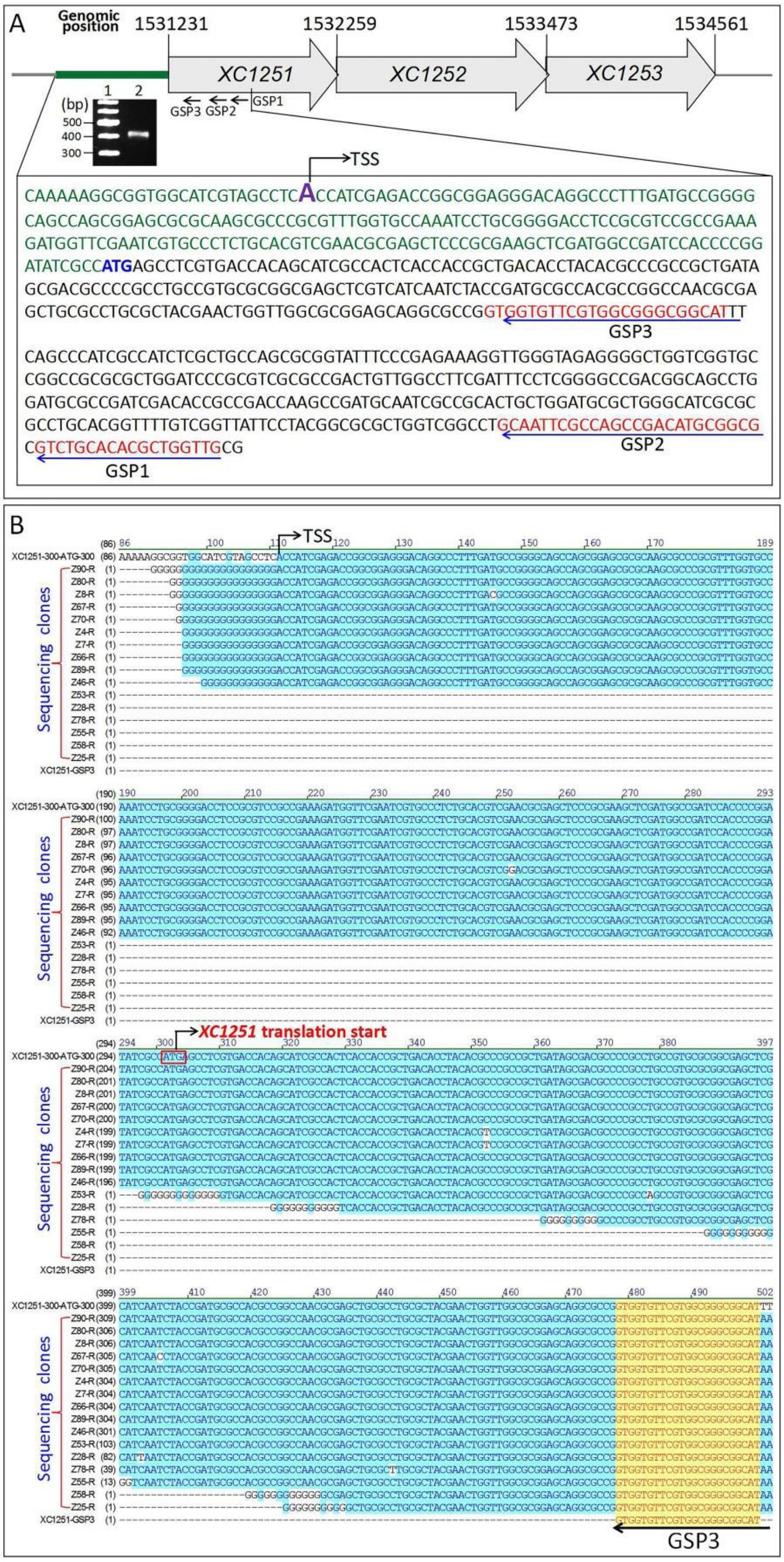

Fig. S1. Identification of the transcription start site (TSS) of the *met* operon (*XC1251*-*XC1253*) by 5'-RACE. **(A)** Schematic representation of the positions and sequences of the primers used for the 5'-RACE, the RACE product (lane 1: DNA marker; line 2: the RACE product) and the identified TSS. **(B)** Alignments of the sequences of the RACE product clones with the flanking sequences of *XC1251* start codon ATG.

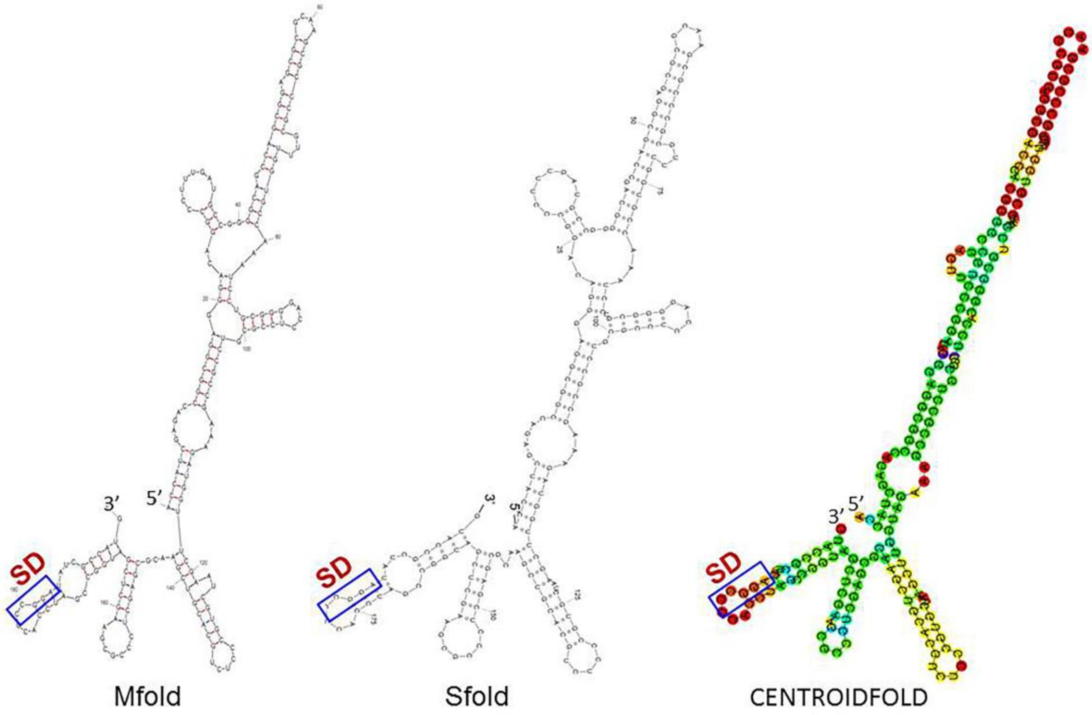

Fig. S2. The potential secondary structure of the 5'UTR of the *Xcc met* operon. The structure was predicted respectively by Mfold (Zuker, M. Mfold web server for nucleic acid folding and hybridization prediction. *Nucleic Acids Res* 2003;31:3406-3415), Sflod (Ding Y, Chan CY, Lawrence CE. Sfold web server for statistical folding and rational design of nucleic acids. *Nucleic Acids Res* 2004;32:W135-141), and CENTROIDFOLD (Sato K, Hamada M, Asai K, Mituyama T. CENTROIDFOLD: a web server for RNA secondary structure prediction. *Nucleic Acids Res* 2009;37 (Web-Server-Issue):277-280).
